# Advances in algorithms for normalizer gene selection in qRT-PCR: implications for cancer biology and precision medicine

**DOI:** 10.3389/fgene.2026.1762055

**Published:** 2026-03-27

**Authors:** Abhay Kumar Pathak, Sukhad Kural, Lalit Kumar, Sumit Saini, Manjari Gupta

**Affiliations:** 1 Department of Computer Science, Institute of Science, Banaras Hindu University, Varanasi, India; 2 Department of Urology, Institute of Medical Sciences, Banaras Hindu University, Varanasi, India; 3 Allegheny Health Network, Pittsburgh, PA, United States

**Keywords:** biomarkers, housekeeping genes, normalization, precision medicine, qRT-PCR

## Abstract

Quantitative Reverse Transcription Polymerase Chain Reaction (qRT-PCR) plays a significant role in gene expression analysis in cancer research and precision medicine. It allows precise quantification of gene expression variation which is necessary for understanding tumor biology, identifying predictive biomarkers and developing therapeutics interventions. However the accuracy and stability of qRT-PCR data heavily rely on finding stable reference genes. The gene stability refers to minimal variation in expression levels of a candidate reference gene across different biological conditions, sample groups and technical replicates. Traditionally, housekeeping genes such as β-actin, GAPDH and 18S rRNA have been used for normalization but consistency and variation can vary under different experimental settings. Over time, mathematical and statistical tools such as geNorm, NormFinder, BestKeeper and gQuant have been developed to find most stable reference genes. These algorithms have become essential in ensuring accurate and reproducible data in cancer research, where gene expression profiles can vary significantly across different tumor types, stages and individual patients. This review focuses on the progression and advancements of traditional and advanced reference gene selection methods, applications in cancer research and their significant role in precision medicine. It presents an overview of the commonly employed normalizers, outlining their respective advantages and limitations, and includes a concise discussion on the assessment of gene stability across diverse experimental contexts. Additionally, it emphasizes their use in cancer research and their importance in enhancing the accuracy and consistency of gene expression normalization, particularly within precision medicine.

## 1 Introduction

Quantitative Reverse Transcription Polymerase Chain Reaction (qRT-PCR) is one of the most widely used methods for quantification of gene expression in biological research (Artika et al., 2022). Due to high sensitivity and specificity it became indispensable for understanding molecular mechanism and gene expression in different contexts including cancer research and precision medicine (Wong and Medrano, 2005). Oncology research in particular has benefitted highly from qRT-PCR as it enables for detection and quantification of gene expression varies which are central to understanding tumor biology, metastasis and treatment (Chen et al., 2019). Even with the high sensitivity and specificity of qRT-PCR data, it must be interpreted carefully because technical changes can introduce significant errors. Changes in RNA quality, reverse transcript stability and PCR amplification are all factors which could impact the accuracy of qRT-PCR data (Bust et al., 2009; Đermić et al., 2023). To address this challenge the need of normalizers becomes essential. Normalizer addresses these potential biases by adjusting the target gene expression data based on the expression stable reference genes. These reference genes are also known as housekeeping genes which have the same expression pattern across different samples conditions and experimental environments. Some commonly used housekeeping genes include GAPDH, β-actin and 18S rRNA, which are responsible for fundamental processes such as metabolism, cytoskeletal structure and ribosomal function, although their stability can vary depending on the biological context (Chapman and Waldenström, 2015; Djari et al., 2024).

The stability of these housekeeping genes has been an area of concern, particularly in cancer studies (Gu et al., 2023). Because tumors are highly heterogeneous, their gene expression patterns can differ widely across tumor types, stages and patient populations. Genes such as GAPDH and β-actin, which are regarded as stable in normal biological systems, may exhibit altered expression in malignant conditions due to the distinct metabolic and structural requirements of rapidly proliferating cells (Dheda et al., 2005). As a result, relying on these reference genes for normalization may provide misleading interpretations, emphasizing the need of selecting reference genes validated for the specific conditions under the particular conditions of a study.

**FIGURE 1:**
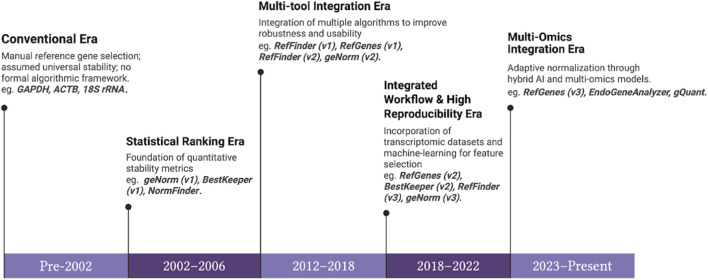
Evolutionary Timeline of Normalizer Gene Selection Methods in qRT-PCR (2002–2025).

The important task is to identify appropriate reference genes for normalisation that has led to the incremental evolution of specialised algorithms and statistical tools as stated in Figure 1. Traditional approaches for stable reference gene selection heavily relied on empirical evidence to validate, Where the researchers and scientists selected stable reference genes based on their presumption for experimental validation and stability of them in various conditions (De Spiegela et al., 2015). Although this approach provides some insights about gene stability, it is time consuming, lacks scalability and labor intensive. In addition to that, empirical selection of stable reference genes are not taking account of potential change in gene expression under different experimental setups like cancerous versus normal tissues.

To overcome these challenges, various statistical algorithms such as geNorm (Vandesompele et al., 2002), NormFinder (Andersen et al., 2004), BestKeeper (Pfaffl, 2004), RefFinder (Xie et al., 2023) and gQuant (Pathak et al., 2024) were developed. These tools relied on statistical measures along with weighted and voting mechanisms to quantify the stability of stable reference genes across different experimental settings and rank them based on their stability of the expression. These methods have greatly improved the selection process providing researchers with more established and reproducible settings to identify the most stable reference genes for their specific experimental setup.

The significance of these tools has been invaluable especially in oncology research where accurate normalization is necessary to recognize reliable biomarkers and understanding tumor biology (Skrzypski, 2008). Even in development of precision medicine strategies selection of reliable reference genes have significant implications. It aims to tailor treatment to the individual patients according to their molecular profile which is directly connected to accurate gene expression analysis. By ensuring the reliability and consistency of the normalizer these tools enable accurate biomarker discovery, improved understanding of tumor biology and also helps in identifying therapeutic targets that are necessary for improving patient outcomes (Paik et al., 2004).

This review presents the evolutionary development of stable reference gene selection methods. It focuses on traditional to advanced approaches that have improved the reliability of qRT-PCR results. We explore statistical and mathematical approaches for qRT-PCR reference gene stability assessment. RNA-seq–based resources are discussed only where they support the pre-selection or validation of candidate reference genes for qRT-PCR normalization. Further, we highlighted various advantages and limitations which occur while utilizing the tools in oncology research and discuss their role in advancing precision medicine, showcasing their impact on omics data expression in oncology.

This mini-review is organized as follows: Section 2 summarizes widely used computational tools and statistical methods for reference gene stability evaluation, with an emphasis on algorithmic assumptions and outputs. Section 3 discusses comparative performance trends and highlights key methodological advancements from traditional pairwise and variance-based methods to integrated and ensemble ranking frameworks. Section 4 outlines emerging directions, including improved reproducibility practices and potential integration of AI-driven approaches, followed by concluding remarks on implications for cancer biology and precision medicine.

## 2 Evolution and comparative analysis of reference genes selection tools

Over time, a number of computational tools and statistical methods developed primarily for qRT-PCR reference gene stability evaluation, including selected transcriptomic resources that assist candidate gene pre-screening. Here, we comparatively evaluate major computational tools for reference gene stability assessment highlighted in Table 1, focusing on their statistical assumptions, performance behavior across experimental contexts and practical suitability. Rather than treating the methods as equivalent, we highlight how differences in modeling strategy, dataset scale and biological variability influence tool performance.

**TABLE 1:** Summary of key computational tools and algorithms developed for reference gene stability assessment and normalization in qRT-PCR and transcriptomic analyses.

Tool	Authors & year	Country	Underlying technique/Algorithm	Platform/Implementation	Data type supported	Advantages	Limitations	Future scope/Updates	References
geNorm (v1)	Vandesompele et al. (2002)	Belgium	Pairwise variation and M-value computation	Excel/qBase^+^	qRT-PCR	Well-documented and reproducible	Assumes least variation stability; not for all tissues	Foundation for modern stability algorithms	Vandesompele et al. (2002)
BestKeeper	Pfaffl et al. (2004)	Germany	CV + SD-based ranking with correlation analysis	Excel	qRT-PCR	Fast and easy for small datasets	Not scalable; less reliable for large studies	Continued use in basic qPCR validation	Pfaffl et al. (2004)
NormFinder	Andersen et al. (2004)	Denmark	Model-based ANOVA variance estimation	Excel Add-in/R	qRT-PCR	Considers intra- and inter-group variation; statistically rigorous	Sensitive to missing data; limited scalability	Adaptation for RNA-Seq and single-cell normalization	Andersen et al. (2004)
geNorm (v2)	Mestdagh et al. (2009)	Belgium	Stability index (M-value) via geNorm	qBase^+^/Excel	qRT-PCR	Compatible with new qRT-PCR instruments; reproducible	Sensitive to co-regulated genes	Integration into cloud-based normalization frameworks	Mestdagh et al. (2009)
RefGenes	Hruz et al. (2011)	Switzerland	Genome-wide microarray expression mining + stability ranking	Web-based (Genevestigator platform)	Microarray/large-scale transcriptome datasets	Condition-specific reference-gene identification; uses large expression databases	Dependent on availability and quality of public transcriptome datasets; identified genes still require qRT-PCR validation	Expansion to RNA-Seq datasets; broader organism coverage; integration into automated high-throughput pipelines	Hruz et al. (2011)
RefFinder (v1)	Xie et al. (2012)	United States of America	Deep-sequencing based small RNA analysis: identifies known and novel plant miRNAs	Next-generation small RNA deep sequencing data analysis via software package	deep-sequencing datasets; includes novel miRNAs and miRNA expression profiles	Supports miRNA expression profiling, miRNA detection and miRNA target prediction from sequencing data	Not suitable for standard qRT-PCR normalization workflows or housekeeping gene reference gene stability analysis	Could be extended to more plant species, integrate with updated genome annotations and improved miRNA databases	Xie et al. (2012)
GenExpA	Hoja-Łukowicz et al. (2022)	Poland	Uses the algorithm from NormFinder to rank candidate reference genes and incorporates removal strategy for the least stable genes	Web-based/open-source software	qRT-PCR data	Uses multiple sample combinations rather than only original sample set; Uses NormFinder but removes least-stable genes	Depends on the set of candidate reference genes supplied	The strategy may be extended to more complex experimental designs to ensure normalization robustness	Hoja-Łukowicz et al. (2022)
RefFinder (v2)	Xie et al. (2023)	China	Integrator of geNorm, NormFinder, BestKeeper (geometric mean ranking)	Web interface	qRT-PCR	Comprehensive results combining multiple algorithms	Limited customization; offline unavailability	Integration with next-gen qPCR systems	Xie et al. (2023)
RGeasy	De Souza et al. (2024)	Brazil	Aggregates previously validated qPCR reference genes; uses RefFinder algorithms (geNorm, NormFinder, BestKeeper, ΔCt) for stability ranking across user-selected conditions	Web-based tool (Laravel backend + MySQL + JavaScript frontend)	qRT-PCR datasets from published validation studies	Allows cross-condition, cross-treatment stability evaluation; saves revalidation time; provides metadata	Depends on previously published reference-gene datasets; cannot discover new RGs from scratch; limited for novel tissues/conditions	Integration of RNA-Seq for *de novo* RG discovery; database expansion; high-throughput automation	De Souza et al. (2024)
EndoGeneAnalyzer	Teixeira et al. (2024)	Brazil	Statistical stability analysis using grouping, outlier filtering, descriptive metrics, and non-parametric ranking to identify stable reference genes	Stand-alone software/application (platform-neutral; published as open-access tool)	qRT-PCR and RNA-Seq gene expression datasets	Structured, statistically rigorous approach to choose reference genes; helps ensure reproducible normalization across diverse experimental conditions	Performance depends on input data quality; limited generalization across tissues; may miss complex non-linear patterns	Integration of ML/DL-based scoring, expansion to multi-omics, and deployment in automated analysis pipelines	Teixeira et al. (2024)
gQuant	Pathak et al. (2024)	India	Multi-statistical ensemble voting classifier integrating variance and correlation metrics	Python/Open-source package	qRT-PCR, RNA-Seq	High accuracy, scalable to large and heterogeneous datasets	Computationally intensive; needs high-performance systems	Integration into AI-driven normalization pipelines and cloud platforms	Pathak et al. (2024)

The development of geNorm marked a major breakthrough in stable reference gene selection for qRT-PCR data normalization. It uses pairwise variation and m-value computation. It identifies the normalizer by comparing the average variation of a gene relative to all other genes in the datasets. The assumption of the geNorm is that genes which have least expressional variability across all samples are the most stable. It acted as a foundational tool for many studies but it has a limitation of assumption of that all the genes are suitable references and comparison will provide the most stable reference (Vandesompele et al., 2002).

BestKeepr, also introduced with the same goal of finding candidate reference genes works on the statistical methods combining the coefficient of variation and standard variation. It is easy to implement in Excel, it helps for rapid computation and ranking the stable reference genes. It was a simple and effective tool but failed to work for bigger datasets because of its limited scalability (Pfaffl et al., 2004).

Development of NormFinder in the similar timeline of geNorm, introduces a model based ANOVA variation estimation approach which accounts for both inter-group and intra-group variation. This framework provides a more robust ranking of stable reference genes along with the easy implementation in Excel and R made it more accessible to the researchers. Although missing data and large dimensional datasets remained as a challenge for this tool (Andersen et al., 2004).

The successor of geNorm version 2, includes the weighted stability index that focuses on variability of input data and reproducibility across different commercial platforms such as qBase^+^ and excel. Despite the advancements, this method remains sensitive to co-regulated genes which can still introduce instability as a result (Mestdagh et al., 2009).

The initial version of RefGenes uses a large scale expression database for mining and ranking to assist in the stable reference gene selection process across species. The web-based interface allows cross-species normalisation but is only limited to human and animal tissue coverage. The intuition behind this tool is to leverage transcriptomic databases for reference gene identification which uses multi-organism expansion (Hruz et al., 2011).

RefFinder was created as an integrative platform that combines all the results from different tools such as geNorm, BestKeepr and Normfinder using a ranking system of geometric mean. It is a stable tool and delivers a comprehensive stability index along with different ranking standards by integrating multiple algorithms. It is highly informative and presents very thorough details about the analysis but lacks the customization, restricts high throughput and automated workflows (Xie et al., 2012).

GenExpA is a recently developed software tool for robust reference-gene selection and normalization of qRT-PCR data across varied experimental models and sample sets. It combines the classical stability-ranking algorithm NormFinder with a novel iterative strategy that progressively removes the least stable candidate reference genes and constructs “daughter models” (subsets of samples) alongside the main experimental model then assesses gene expression coherence across all models using a coherence score (CS). This dual-filtering approach helps guard against misleading normalizers that might seem stable in a single dataset, improving the reliability of normalization, reducing bias from unstable housekeeping genes, and ensuring biologically consistent conclusions. GenExpA is open-access, platform-neutral and suitable for standard qRT-PCR datasets offering a more rigorous, statistically-grounded alternative to the simple ‘lowest stability value’ selection of reference genes commonly used in gene-expression studies (Hoja-Łukowicz et al., 2022).

RefFinder version 2, improved with an enhanced integrative ranking method which optimizes the weights of the results from multiple stability metrics. It offers increased stability and robustness to complex experimental setups. However it does not support the RNA-seq datasets analysis, primarily limited to qRT-PCR data analysis (Xie et al., 2023).

RGeasy is a newly developed computational framework designed to simplify and automate reference gene selection for qRT-PCR normalization across diverse biological and experimental conditions. It integrates multiple stability assessment approaches, including geNorm-type pairwise variation, NormFinder-style variance modeling, coefficient-of-variation analysis, and non-parametric ranking strategies to provide robust and unbiased stability evaluation. The tool reduces normalization errors caused by context-dependent fluctuations in housekeeping genes and enables researchers to identify the most stable reference genes with high accuracy. RGeasy offers a streamlined interface with easy data import, interactive visualizations, and automated reporting, making it suitable for both beginners and advanced users. It is freely available, platform-independent, and supports datasets derived from qRT-PCR as well as high-throughput transcriptomic experiments (De Souza et al., 2024).

EndoGeneAnalyzer is a recently developed statistical framework, used for stable reference gene selection across different experimental conditions. It includes multiple classical stability assessment scenarios such as group analysis, outlier sensitivity, detailed descriptive statistics and non-parametric ranking tests to assess stability of gene expression. It overcomes bias arising from variable expression patterns and provides most stable reference genes. It is available in open access and platform neutral which can support qRT-PCR and RNA-Seq datasets (Teixeira et al., 2024).

The most recent invention in the field, gQuant incorporates a multi-statistical ensemble voting classifiers that integrates multiple statistical methods such as geometric mean, covariance standard deviations and kernel density estimation (Pathak et al., 2024). It also scales the gene expression data to be insensitive to the outliers and provides the remedies for missing values imputations. The source code is open access written in a python package accessible to everyone (Pathak et al., 2025). It supports qRT-PCR and RNA-Seq datasets which offers high stability and scalability to large scale heterogeneous datasets.

### 2.1 Comparative practical insights

Pairwise comparison–based methods such as **geNorm** perform reliably in small, well-controlled datasets but can falsely rank co-regulated genes as stable due to their assumption of expression independence. Variance-modeling approaches like **NormFinder** are more robust in heterogeneous cancer datasets because they account for intra- and inter-group variability. **BestKeeper** is practical for rapid screening but loses reliability in large-scale or noisy datasets. Database-driven tools such as **RefGenes** depend on public transcriptomic resources and therefore require experimental validation before qRT-PCR use. Integrative ranking frameworks (**RefFinder, RGeasy**) reduce bias by combining algorithms but inherit the assumptions of underlying tools. Ensemble and machine-learning–oriented tools such as **gQuant** demonstrate improved scalability and robustness to outliers, making them suitable for heterogeneous or high-throughput datasets, although at the cost of computational demand. These distinctions indicate that tool selection should be dataset-driven rather than universal.

For small experimental sets with limited variation, geNorm or BestKeeper remain appropriate. For studies involving multiple biological groups, tumor heterogeneity, or treatment conditions, NormFinder or GenExpA provide more reliable modeling. Integrative tools (RefFinder, RGeasy) are useful when consensus ranking is desired, while ensemble frameworks such as gQuant are better suited for large, multi-condition or multi-omics studies. Database-mining tools should be treated as *pre-screening* systems rather than final selectors.

## 3 Application of reference gene selection in cancer research

In the context of improving rigor and reproducibility in qRT-PCR studies, the updated MIQE 2.0 guidelines place greater emphasis on the careful validation of reference genes rather than assuming the stability of conventional housekeeping genes. Within this framework, computational stability assessment tools play an important supporting role, as they provide objective statistical evidence to guide and justify reference gene selection. The standard approach to ensure accuracy in qRT-PCR experiments is the use of reference or housekeeping genes for normalization, allowing consistent comparison of gene expression across samples (Hannemann et al., 2025). To improve reproducibility and reporting standards in such analyses, the Minimum Information for Publication of Quantitative Real-Time PCR Experiments (MIQE) guidelines were developed and later updated as MIQE 2.0. Since accurate normalization depends on efficiency-corrected quantification and transparent selection of stable reference genes, MIQE 2.0 emphasizes that relying only on raw Cq values is insufficient. However, it is frequently unreliable to assume that these genes exhibit consistent expression across various cancer types, stages and experimental conditions. This indicates the increasing significance of algorithmic approaches in enhancing reference gene selection (Bustin et al., 2025). Stable reference genes give researchers the ability to adjust for technical variation, resulting in more accurate data that can be utilized to assess therapeutic targets, discover biomarkers and examine tumor biology.

### 3.1 Biomarker discovery

Reliable biomarker discovery in cancer research depends on the accurate selection of stable reference genes in qRT-PCR. For example, Hu et al. found that YWHAZ and B2M provided more consistent normalization than traditional controls in colorectal cancer, highlighting cell-line heterogeneity (Hu et al., 2023). HNRNPL, IPO8 and PUM1 were also found to be more stable than GAPDH or ACTB in a pan-cancer evaluation studied by Rácz (Goossens) et al. in a variety of cancer models (Rácz et al., 2021). Ahn et al. found that in hepatocellular carcinoma tissues and matched blood samples, HMBS was the most stable reference gene, compared to commonly used controls (Ahn et al., 2021). Gorji-Bahri et al. revealed that ACTB/HPRT1/B2M was stable in hepatic cells and YWHAZ/UBC was stable in breast cancer, further demonstrating the variations in stability across tissues (Gorji-Bahri et al., 2021). Variation in the expression of conventional housekeeping genes like GAPDH and ACTB across cancer types highlights the context-dependent nature of reference gene stability and its impact on accurate normalization in qRT-PCR (Jo et al., 2019). Such findings highlight the need for algorithm-based approaches that can objectively identify context-specific reference genes to ensure reliable biomarker interpretation.

### 3.2 Tumor heterogeneity

Gene expression often fluctuates across different regions of a tumor and this heterogeneity makes achieving consistent normalization in qRT-PCR particularly challenging. Differences in reference gene stability may arise not only among cancer types but also between distinct tumor regions and the diverse cell types present within the tumor (Nevone et al., 2023). For example, James et al. observed that in oral squamous cell carcinoma, ACTB and TBP showed variable expression between lymph-node and stromal samples, whereas RPLP0 and 18S rRNA remained relatively stable across tissues (James et al., 2024). These findings indicate that tumor development and microenvironmental diversity can influence reference gene behavior, highlighting the need to consider heterogeneity when interpreting qRT-PCR data in cancer studies.

### 3.3 Therapeutic target validation

Validating possible therapeutic targets is an essential step in the development of new drugs for cancer treatment. Reliable qRT-PCR normalization is necessary for accurate therapeutic target validation in order to differentiate between experimental variability and actual drug-induced gene expression changes. For example, Gu et al. reported that in lung cancer cells exposed to hypoxia and serum deprivation, GAPDH and ACTB fluctuated by 20%–75%, whereas CIAO1, CNOT4 and SNW1 remained stable and improved normalization accuracy (Gu et al., 2023). Similarly, Iskhakova et al. found that mTOR-inhibitor–induced dormancy caused instability in ACTB, RPS23 and RPL13A, while B2M and YWHAZ were consistently reliable (Iskhakova et al., 2025). Pan-cancer analysis also suggests that conventional genes like GAPDH and ACTB exhibit considerable variability, whereas genes like IPO8, PUM1 and RPLP1 have improved stability (Rácz et al., 2021; Malcolm et al., 2025). These results show that drug efficacy measurements can be altered by the use of unvalidated reference genes.

### 3.4 Prognostic and predictive models

Reference genes are essential in developing prognostic and predictive models in cancer research. In pediatric gliomas, Hernández-Ochoa et al. showed that PKM and TPI1 were the most stable reference genes and normalization with PKM revealed grade-associated increases in glycolytic genes such as HK1 and PFKM (Hernández-Ochoa et al., 2021). Similarly, in HER2-enriched breast cancer cells, Jain et al. revealed that reference-gene triplets including HSP90AB1, DAD1, PFN1 and PUM1 perform better than conventional controls like ACTB or GAPDH, strengthening subtype-specific classifiers (Jain et al., 2021). Clinical multigene assays, such as the OncoMasTR 6-gene test for ER^+^/HER2^-^ for early breast cancer, emphasize the importance of proper normalization for comparable risk assessment across laboratories (Loughman et al., 2022). However, some recent prognostic markers still rely on a single housekeeping gene, such as β-actin, which shows how hidden variability can be introduced by unvalidated reference genes (Wu et al., 2024). Together, these findings show that reliable prognostic and predictive models require validated and often multi-gene normalization strategies.

## 4 Application of reference gene selection in precision medicine

Although RNA-seq studies contribute to identifying stable gene candidates, final validation and normalization in clinical workflows continue to rely on qRT-PCR, which remains the focus of this review. The need for proper reference gene selection is growing in precision oncology, since modern multi-OMICS techniques such as RNA-seq, proteogenomics, metabolomics and single-cell transcriptomics still require qRT-PCR to validate essential molecular findings. TCGA RNA-seq data from 12 different tumor types were analyzed pan-cancer by Krasnov et al. proved that no single housekeeping gene is consistently stable and found the best pairs of reference genes for each kind of cancer, highlighting the necessity of context-specific normalizers in extensive transcriptome research (Krasnov et al., 2019). Razavi et al. evaluated 12 potential reference genes at the tissue level in 45 paired papillary thyroid carcinoma, adjacent normal and multinodular goiter samples. Using geNorm, NormFinder and BestKeeper, they discovered that GUSB and HPRT1 were the most stable controls, demonstrating how algorithm-guided selection enhances clinical qRT-PCR protocols (Razavi et al., 2019). Liquid biopsy based OMICS studies demonstrate similar needs for stable reference markers. Wen et al. analyzed platelet RNA-seq from a pan-cancer cohort to derive 95 candidate reference genes and validated several by RT-qPCR for early cancer detection (Wen et al., 2022). Dai et al. similarly integrated RNA-seq and miRNA-seq of serum exosomes to identify OAZ1, hsa-miR-6835–3p and multi-gene panels as stable normalizers, showing low expression variability and validation in ovarian cancer samples (Dai et al., 2021). Together, these studies show that reference gene stability is not uniform across samples, highlighting the need for systematic and data-driven selection to ensure reliable qRT-PCR validation in tissue and liquid biopsy OMICS workflows.

## 5 Current challenges and future directions

Despite major advancements in stable reference gene selection tools, several challenges still hinders the accuracy and consistency of qRT-PCR based gene expression studies related to oncology and precision medicine. The lack of a universal reference gene is still the most significant challenge. No reference gene is consistently stable under all conditions and even small Cq shifts can lead to significant errors in expression fold-change (Hwang et al., 2025; Schmittgen and Livak, 2008). In cancer, heterogeneity, therapeutic effects and metabolic changes can also affect the stability of reference genes. DNA contamination, pseudogene signals, splice variation and inconsistent RNA quality continue to alter experimental results. When combined with variable MIQE reporting and small gene panels, these issues make stability assessments less accurate across studies. Although there are many tools available in the market such as geNorm, BestKeeper, gQuant which have strengthened analytical practice, no single methods or approach are reliable in identifying stable reference gene selection across all various sample types and experimental conditions. The variability in result complicates the comparison between cross-studies and affects the interpretation of results. The tools have also presented difficulties when dealing with different populations from diverse genetic backgrounds, disease types and uneven exposure of the environment, requiring validation for each experimental context. While recent developments have shown multiple ways to deal with missing data, high-throughput and supported multi-omics platforms which are increasingly common in modern cancer studies, still struggle to overcome these challenges in real-time experiments.

Future advances in reference gene normalization will be dependent on adaptable, data-driven approaches capable of dealing with the heterogeneity, scale and molecular complexity of modern cancer research. Artificial intelligence (AI) and ML approaches will improve reference gene discovery by using large-scale datasets to identify multi-gene normalizer sets optimized for specific tissues, tumor subtypes and experimental platforms (Cheng et al., 2024). These methods are likely to facilitate the shift from single housekeeping genes to multi-gene reference panels suited to specific experimental conditions (Rácz et al., 2021). As newer methods like single-cell qPCR and liquid biopsies produce very limited and variable RNA, normalization tools must be improved to handle these differences and still identify stable reference genes (Cuevas-Diaz Duran et al., 2024). Automated pipelines that align with revised MIQE guidelines, incorporate batch correction and missing-data handling and operate on cloud-based platforms will facilitate more reproducible analyses across laboratories and multi-center research (Bustin, , 2025; Hampton et al., 2025). Enhanced computational tools should address these challenges, incorporate stability into gene selection ranking and account for diversity across different populations (Jóźwik et al., 2025).

## 6 Conclusion

Since molecular variability is high in precision medicine and cancer research, accurate normalization remains a crucial factor in determining qRT-PCR reliability. Over time, the research has evolved from using traditional housekeeping genes and toward more structured and evidence-based techniques to finding appropriate reference genes. Ongoing concerns such as tumor heterogeneity, fluctuating sample quality and inconsistent reporting standards underline the need for more comprehensive validation strategies that include different data sources. Establishing MIQE-aligned, well-standardized procedures together with the integration of multi-omics information, larger datasets and ML supported stability evaluation methods will help strengthen reproducibility. A robust selection of reference genes will improve biomarker identification, therapeutic assessment and the development of patient-specific clinical applications.
